# Interictal burden in migraine: a systematic review of conceptual domains and measurement properties of instruments

**DOI:** 10.1186/s10194-026-02458-0

**Published:** 2026-07-23

**Authors:** Cornelius Angerhöfer, Yutaro Fuse, Samuel Van Langenhove, Thomas Canning, Alice C. von Alvensleben, Sheharyar Ahmad, Ane Murillo, Laia Boada, Maria M. Roque, Antoinette MaassenVanDenBrink, Christian Lampl

**Affiliations:** 1 Department of Neurology with Experimental Neurology, Charité - Universitätsmedizin Berlin, Corporate Member of Freie Universität Berlin and Humboldt-Universität zu Berlin, Berlin, Germany; 2 Department of Artificial Intelligence Medicine, Chiba University Graduate School of Medicine, Chiba, Japan; 3 Department of Neurology, Ghent University Hospital, Ghent University, Ghent, Belgium; 4 Department of Psychological Medicine, Institute of Psychiatry, Psychology, and Neuroscience (IoPPN), King’s College London, London, UK; 5 Wolfson Sensory, Pain and Regeneration Centre, IoPPN, King’s College London, London, UK; 6 Institute of Public Health (IPH), Charité - Universitätsmedizin Berlin, Corporate Member of Freie Universität Berlin and Humboldt-Universität zu Berlin, Berlin, Germany; 7 Department of Psychology, Università degli studi della Campania Luigi Vanvitelli, Caserta, Italy; 8 Neurology Department, Vall d’Hebron University Hospital, Barcelona, Spain; 9 Neurology Department, Hospital de Santa Maria, ULS Santa Maria, Lisboa, Portugal; 10 Division of Vascular Medicine and Pharmacology, Department of Internal Medicine, Erasmus MC, University Medical Center Rotterdam, Rotterdam, the Netherlands; 11 Hospital Barmherzige Brüder Linz, Linz, Austria

**Keywords:** Migraine, Interictal burden, Patient-reported outcome measures, COSMIN, Psychometric properties

## Abstract

**Background:**

Migraine imposes significant burden not only during acute attacks but also during headache-free interictal periods. Several patient-reported outcome measures (PROMs) have been used to assess this interictal burden, yet the quality of their measurement properties has not been systematically evaluated.

**Objectives:**

To identify instruments used to assess interictal burden in migraine, evaluate their measurement properties using the COSMIN methodology, and synthesize the conceptual domains of interictal burden.

**Methods:**

A systematic search of MEDLINE, Embase, PsycINFO, CINAHL, and Web of Science Core Collection was conducted from database inception to 20 February 2026. Studies evaluating measurement properties of PROMs assessing interictal burden or migraine impact in migraine populations were eligible. The COSMIN risk-of-bias checklist and criteria for good measurement properties were applied. Findings were synthesized in accordance with PRISMA 2020.

**Results:**

Among 29 included studies, three instruments were identified: the Migraine Interictal Burden Scale-4 (MIBS-4; 26 studies), the Eurolight questionnaire (1 study), and the Fear of Attacks Inventory (FAMI; 2 studies). Seven conceptual domains of interictal burden were identified: functional impairment, social and relational impact, psychological and emotional distress, cognitive dysfunction, sensory symptoms, physical symptoms, and behavioral modifications. Evidence of measurement properties was concentrated in construct validity (19 studies) and responsiveness (8 studies). Structural validity, internal consistency, test–retest reliability and content validity were sparsely evaluated. The MIBS-4 demonstrated consistent convergent and known-groups validity and sensitivity to change across preventive therapies, including CGRP-targeted treatments and onabotulinumtoxinA.

**Conclusions:**

The MIBS-4 is the most widely used instrument for assessing interictal burden. Despite encouraging evidence of construct validity and responsiveness, comprehensive psychometric studies addressing content and structural validity, reliability, and measurement error, alongside conceptual work towards broader interictal burden assessment, are needed.

**Registration:**

PROSPERO CRD420261296323; **Clinical trial number**: not applicable.

**Supplementary Information:**

The online version contains supplementary material available at 10.1186/s10194-026-02458-0.

## Introduction

Migraine is a major contributor to global disability [1], imposing a substantial burden on both individuals’ quality of life and on society as a whole [2]. While its most characteristic and well-known feature is recurrent, unilateral headache attacks, it is well recognized that migraine is a cyclical disorder encompassing four phases: interictal, preictal, ictal, and postictal [3]. Traditionally, both research and clinical management have focused on the ictal phase, i.e. the period of headache pain, as the primary target for reducing migraine burden [4]. However, growing evidence suggests that the burden of migraine extends beyond the acute headache phase [5–7].

The term interictal burden refers to the range of impairments experienced by individuals with migraine during headache-free periods. These impairments may include cognitive difficulties, emotional distress (e.g., anxiety or anticipatory fear of attacks), sensory sensitivities, and limitations in social and occupational functioning [8–10]. Collectively, these factors contribute to a continuous and often underrecognized disease burden, even in the absence of acute headache symptoms.

Despite increasing recognition of interictal burden, its assessment remains limited and inconsistent. Most patient-reported outcome measures (PROMs) used in migraine research focus either on the ictal phase—such as the Migraine Disability Assessment (MIDAS) and the Headache Impact Test (HIT-6)—or on overall quality of life, as measured by the 36-Item Short Form Health Survey (SF-36) and the Migraine-Specific Quality of Life Questionnaire (MSQ). Instruments specifically developed to assess interictal burden are scarce, vary in their conceptual scope, and have not yet been systematically evaluated using contemporary methodological standards.

This systematic review aims to identify, evaluate, and synthesize the available evidence on instruments used to assess interictal burden in migraine. Specifically, the review will (1) catalogue available PROMs and clinician-administered instruments, (2) identify and characterize their conceptual domains and (3) evaluate their measurement properties using the Consensus-based Standards for the Selection of Health Measurement Instruments (COSMIN) methodology [11]. The COSMIN approach provides a rigorous framework for evaluating the quality of outcome measurement instruments, including their validity, reliability, and responsiveness.

## Methods

This systematic review was conducted in accordance with COSMIN methodology and is reported following the Preferred Reporting Items for Systematic Reviews and Meta-Analyses (PRISMA) statement [12]. The review protocol was pre-registered in PROSPERO 2026 (CRD420261296323).

### Selection and screening

A comprehensive literature search was conducted in MEDLINE (via PubMed), Embase (via Ovid), PsycINFO (via Ovid), CINAHL (via EBSCO) and Web of Science Core Collection from database inception up to 20 February 2026. The search strategy combined three key components in line with COSMIN recommendations: (1) terms related to interictal burden (e.g., “interictal,” “between attacks,” “headache-free, “non-headache days”, “fear of next attack”) (2) terms related to the population (migraine, including controlled vocabulary such as MeSH and Emtree as well as free-text synonyms), and (3) a highly sensitive COSMIN-recommended filter for identifying studies on measurement properties (e.g., “instrument,” “psychometr,” “clinimetr,” “validation,” “reliability,” “validity”) [13]. Additional searches included backward citation searching of reference lists of included studies and relevant reviews and forward citation tracking using Web of Science. The search strategy was developed in consultation with a medical librarian and peer-reviewed using the PRESS checklist [14]. All retrieved records were exported to a reference management system. Automatic and manual deduplication was performed using combinations of title, first author, publication year, and unique identifiers such as DOI or PMID. Three reviewers (TC, SVL, CA) independently screened titles and abstracts, followed by full-text articles for eligibility. Any discrepancies were resolved through discussion and consensus.

### Inclusion and exclusion criteria

Studies were considered eligible if they included adults and/or children and adolescents diagnosed with migraine, either episodic or chronic, according to the International Classification of Headache Disorders, 3rd edition (ICHD-3) or earlier [15]. Studies with mixed headache populations were eligible for inclusion if migraine-specific data were reported separately or could be extracted. Studies focusing exclusively on non-migraine primary headache disorders, secondary headaches, or medication-overuse headache without migraine-specific data were excluded.

Eligible studies included instrument development studies describing the creation and initial validation of measures, cross-cultural adaptation and validation studies and studies evaluating one or more measurement properties. Narrative reviews, editorials, commentaries, conference abstracts with insufficient methodological detail, systematic reviews and case reports or case series were excluded.

### Data extraction and analysis

Data extraction was conducted independently by three reviewers (CA, SA, MMR) using a standardized extraction form, with independent verification by two additional reviewers (YF, AD). Extracted data included study characteristics (author, year, country, study design, sample size, and setting), population characteristics (age, sex, migraine type, and diagnostic criteria), instrument characteristics (name, version, language, number of items, domains, and scoring method), measurement property results (including statistical estimates and authors’ interpretations). Any disagreements were resolved through discussion among the five reviewers (CA, SA, MMR, YF, AD).

The primary outcomes of interest were the measurement properties of instruments assessing interictal burden in migraine, evaluated in accordance with the COSMIN taxonomy. The following properties were assessed: content validity (including face validity), structural validity, internal consistency, reliability, measurement error, criterion validity, construct validity (hypotheses testing), cross-cultural validity, and responsiveness. For construct validation, reported associations between interictal burden measures and related constructs were evaluated using Pearson’s (*r*) or Spearman’s (*ρ*) correlation coefficients. Correlation coefficients range from − 1 to + 1, with absolute values of 0.00–0.29 considered weak, 0.30–0.49 moderate, 0.50–0.69 good, and ≥ 0.70 strong.

The methodological quality of each study for each measurement property was evaluated using the COSMIN Risk of Bias checklist, applying the “worst score counts” principle (ratings: very good, adequate, doubtful, or inadequate). The results of each study were evaluated against the updated COSMIN criteria for good measurement properties (version 2.0, 2024) and rated as sufficient (+), insufficient (−), inconsistent (±), or indeterminate (?). Ratings were classified as indeterminate (?) when required information was missing or inappropriate methods were used. When multiple studies assessed the same property, results were qualitatively synthesized and summarized per instrument.

The quality of the body of evidence for each measurement property was graded using the COSMIN-modified GRADE approach, considering the domains of risk of bias, inconsistency, imprecision, and indirectness, and was categorized as high, moderate, low, or very low quality, starting from high-quality evidence and downgraded as appropriate. Inconsistency was considered when results across studies were conflicting (e.g., both sufficient and insufficient findings) and could not be explained by differences in populations or methods. Imprecision was primarily assessed based on the total sample size across studies (downgraded by one level when the total sample size was below 100 and by two levels when it was below 50).

In line with COSMIN recommendations for systematic reviews of measurement properties, a meta-analysis was not conducted, as pooling psychometric estimates across heterogeneous populations, study designs, and statistical methods is generally inappropriate. Instead, findings were synthesized and presented in summary tables.

Additionally, a conceptual framework of interictal burden domains was developed through inductive content analysis of three sources: (1) instrument item content and domain structures, (2) explicit definitions of interictal burden provided by study authors, and (3) study findings describing burden-related outcomes. Two reviewers (CA, YF) independently coded all relevant concepts and grouped them into higher-order domains, with disagreements resolved by discussion. For study-level domain mapping, we used a two-layer coding system. A check mark indicated that study authors explicitly defined, discussed, or analysed the domain as a component of interictal burden. An open circle indicated that the domain was covered by the item content of the instrument used but was not individually discussed by the study authors. The cognitive dysfunction domain was reserved for studies explicitly addressing cognitive ability, concentration, memory, or cognitive processing; therefore, the broad MIBS-4 item on “emotional and cognitive distress” was mapped to psychological/emotional distress and was not automatically coded as cognitive dysfunction unless cognitive symptoms were separately discussed.

## Results

### Study selection

**Fig. 1:**
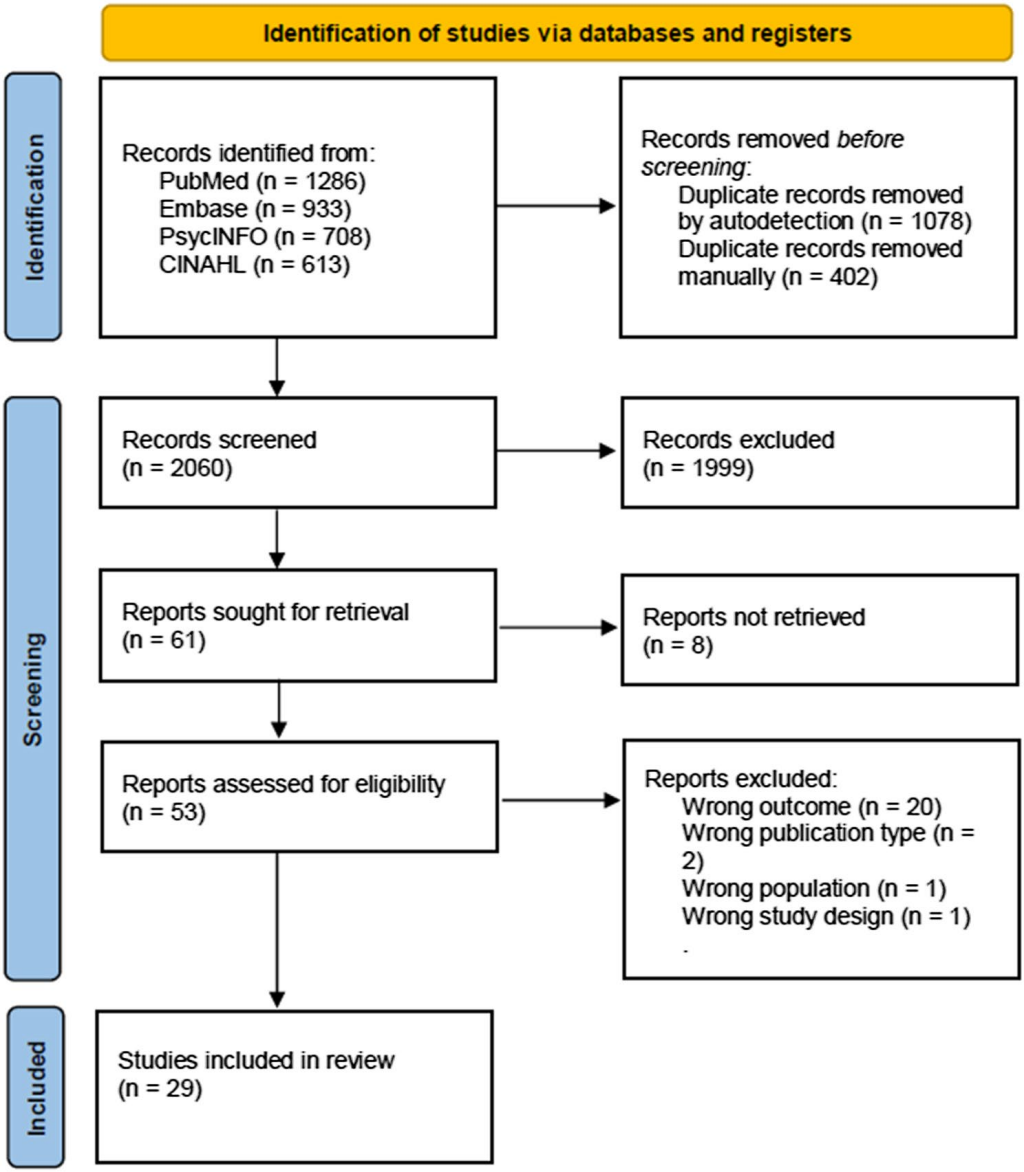
PRISMA 2020 flow diagram

The systematic search of MEDLINE (PubMed), Embase (Ovid), PsycINFO (Ovid), CINAHL (EBSCO), and Web of Science Core Collection, conducted from database inception up to 20 February 2026, yielded 3540 records. After removal of 1480 duplicates, 2060 unique records underwent title and abstract screening. Of these, 61 records were selected for full-text review. Following full-text assessment against the predefined eligibility criteria, 29 studies were included in the final synthesis. The main reasons for exclusion at the full-text stage were absence of measurement property data, use of instruments outside the scope of this review, and non-migraine populations. The complete study selection process is presented in Fig. 1 (PRISMA 2020 flow diagram).

### Study characteristics

The 29 included studies were published between 2007 and 2025 (Table 1). A large number of studies was published in recent years: 20 of 29 studies (69%) were published in 2024 or later, and only two studies predated 2020. Of the 29 publications (Table 1), 26 were full peer-reviewed papers and three were conference abstracts. Study designs comprised cross-sectional or observational surveys (*n* = 20), randomized controlled trials (*n* = 4), instrument validation or adaptation studies (*n* = 4) and one longitudinal observational study.

**Table 1:** Characteristics of included studies

Author (Year)	Study Design	Sample Size	Mean Age	Female %	Migraine Type	Instrument(s)	Publication Type
Argyriou et al. (2024)	Prospective observational (open-label) study	70	44.4	91.5	CM	MIBS-4	Full paper
Awaki et al. (2024)	Cross-sectional observational study	17,071	40.7	66.5	EM + CM	MIBS-4	Full paper
Barbanti et al. (2024)	Prospective observational (real-world, multicenter) study	44	49.7	81.8	EM + CM	MIBS-4	Full paper
Barbanti et al. (2025a)	Prospective observational (real-world, multicenter) study	215	47.9	73	EM + CM	MIBS-4	Full paper
Barbanti et al. (2025b)	Prospective observational (real-world, multicenter) study	82	45.3	91.5	EM + CM	MIBS-4	Full paper
Buse et al. (2007)	Instrument development and validation study	NR	NR	NR	NR	MIBS-4	Conference abstract
Evers et al. (2024)	Cross-sectional observational study	20,756	40.4	60.3	EM + CM	MIBS-4	Full paper
Garcia-Azorin et al. (2020)	Randomized controlled trial	462	NR	NR	EM + CM	MIBS-4	Conference abstract
Hashimoto et al. (2025)	Cross-sectional observational study	81	46	79	EM + CM	MIBS-4	Full paper
Hubig et al. (2022)	Cross-sectional survey study	506	NR	NR	EM + CM	MIBS-4	Full paper
Igarashi et al. (2023)	Cross-sectional observational study	17,071	40.7	66.5	EM + CM	MIBS-4	Full paper
Karaci et al. (2024)	Validation study	178	39.1	87.4	EM + CM	MIBS-4	Full paper
Kaske et al. (2025)	Cross-sectional observational study	46	33.8	80.4	NR	FAMI	Full paper
Klan et al. (2022)	Instrument development and validation study	387	40.9	94.1	EM + CM	FAMI	Full paper
Lampl et al. (2016)	Cross-sectional observational study	6,455	18–65 (range)	62.1	EM + CM	Eurolight questionnaire	Full paper
Lampl et al. (2024)	Cross-sectional observational study	500	40.6	90	EM + CM	MIBS-4	Full paper
Lipton et al. (2023)	Randomized controlled trial	462	NR	NR	EM + CM	MIBS-4	Full paper
Malheiro et al. (2025)	Validation study	459	45.1	93.7	EM + CM	MIBS-4	Full paper
Matsumori et al. (2022)	Cross-sectional observational study	17,071	40.7	66.5	EM + CM	MIBS-4	Full paper
Pascual et al. (2023)	Cross-sectional observational study	10,229	NR	NR	EM + CM	MIBS-4	Full paper
Pozo-Rosich et al. (2025)	Randomized controlled trial	652	NR	NR	EM	MIBS-4	Full paper
Sánchez-Huertas et al. (2025)	Prospective observational study	150	44	91.3	CM	MIBS-4	Full paper
Sandoe et al. (2021)	Randomized controlled trial	462	NR	NR	EM + CM	MIBS-4	Conference abstract
Sarkar et al. (2025)	Cross-sectional observational study	6,267	41.5	90.8	EM + CM	MIBS-4	Full paper
Shapiro et al. (2024)	Cross-sectional observational study	59,001	41.3	74.9	EM + CM	MIBS-4	Full paper
Sotero et al. (2025)	Cross-sectional observational study	200	44.8	92	EM + CM	MIBS-4	Full paper
Takizawa et al. (2025a)	Cross-sectional observational study	19,590	NR	NR	EM + CM	MIBS-4	Full paper
Takizawa et al. (2025b)	Cross-sectional observational study	9,766	36.8	100	EM + CM	MIBS-4	Full paper
Vernieri et al. (2025)	Prospective observational (real-world, multicenter) study	106	50.6	87.7	EM + CM	MIBS-4	Full paper

EM, episodic migraine; CM, chronic migraine; NR, not reported

Sample sizes ranged from 44 to 59,001, with a median of 462. Several studies drew from large population-based cohorts: the OVERCOME (Observational Survey of the Epidemiology, Treatment, and Care of Migraine) program contributed data from Japan (*n* = 17,071 and *n* = 19,590 in two survey waves), the United States (*n* = 59,001 cross-sectional; *n* = 11,634 longitudinal), the European Union (*n* = 20,756), and Spain (*n* = 10,229). Clinical samples ranged from 44 (EMBRACE trial) to 6,267 (HeAD-US study).

The majority of studies (*n* = 24) included both episodic migraine (EM) and chronic migraine (CM) populations. Two studies focused exclusively on CM [16, 17], two included only EM [18, 19] and one examined a mixed population of migraine and tension-type headache within the Eurolight project [20]. Geographically, studies were conducted across multiple regions. Japan contributed the largest number of publications through the OVERCOME Japan surveys. European studies included cohorts from Portugal, Turkey, Greece, Italy, Spain, Austria, and a multinational European sample. North American data were provided principally through the OVERCOME US survey and the CONQUER randomized trial.

### Identified instruments

Three distinct instruments assessing interictal burden were identified across the 29 included studies.

#### Migraine Interictal Burden Scale-4 (MIBS-4)

The MIBS-4 was by far the most frequently used instrument, employed in 26 of 29 studies (90%). It is a four-item self-report questionnaire assessing the burden of migraine between attacks over the past four weeks across four domains: (1) impairment at work or school, (2) impairment in family and social life, (3) difficulty making plans or commitments, and (4) emotional and cognitive distress. Each item is scored from 0 to 3, yielding a total score of 0–12, with established cutoffs for no (0), mild (1–2), moderate (3–4), and severe (≥ 5) interictal burden [21].

#### Eurolight questionnaire

One study [20] used interictal burden questions embedded within the Eurolight questionnaire, a comprehensive cross-sectional survey instrument designed for the Eurolight project across ten European countries. This questionnaire assesses interictal symptoms including anxiety, avoidance behavior, and cumulative burden, but does not yield a single interictal burden summary score.

#### Fear of Attacks in Migraine Inventory (FAMI)

Two studies [19, 22] used the FAMI, containing 29 questions, to assess anticipatory anxiety and fear of attacks as central components of interictal burden in migraine.

### Conceptual domains of interictal burden

**Fig. 2:**
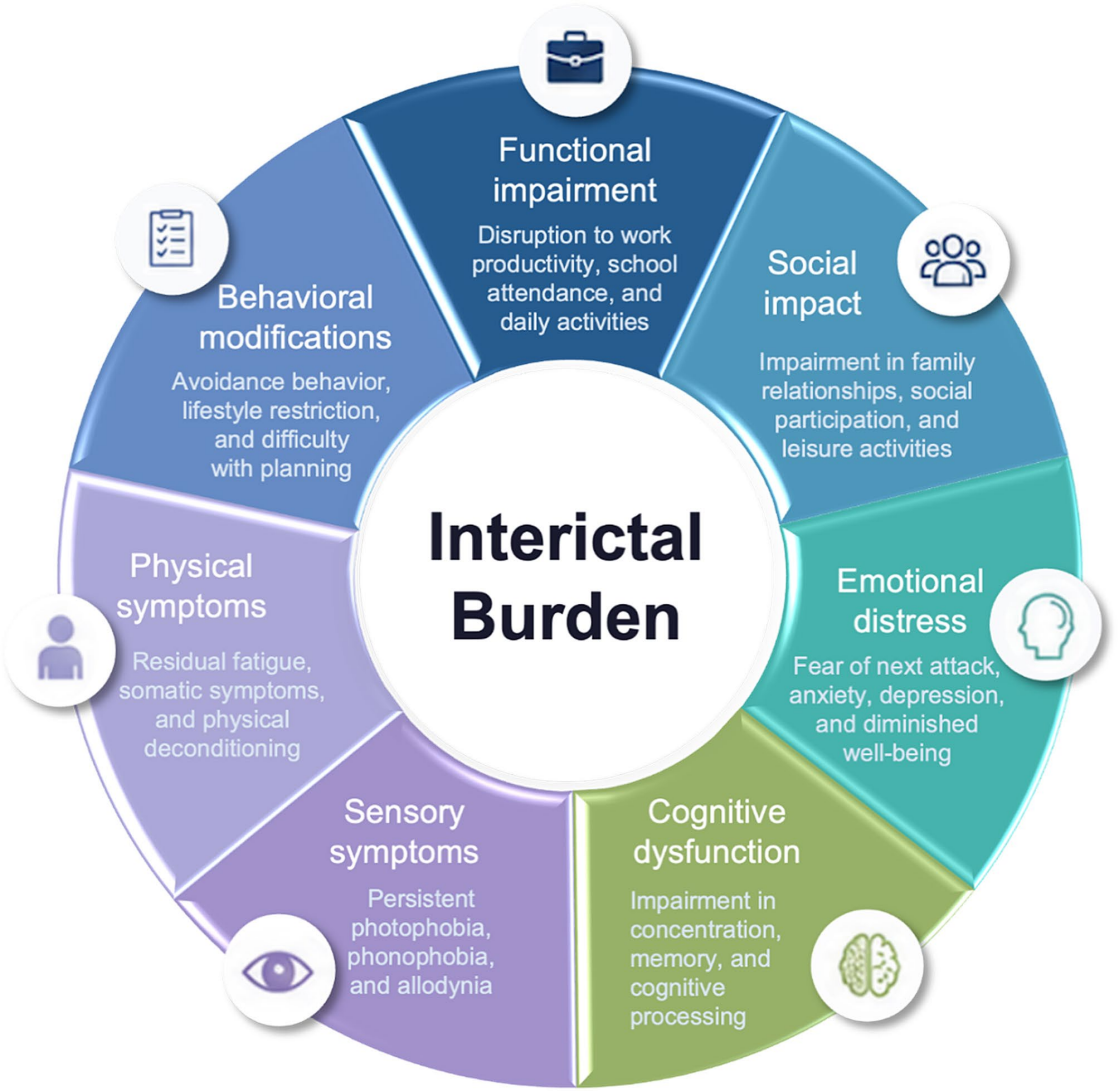
Conceptual domains of interictal burden

Of the 29 included studies, 21 (72%) provided an explicit definition of interictal burden or described its constituent domains (Supplementary Table 1). Across studies, interictal burden was consistently conceptualized as the impact of migraine between attacks, encompassing impairments in daily functioning, quality of life, and psychosocial well-being. Definitions commonly emphasized disruptions in work or school performance, social and family life, emotional distress, and difficulties in planning, while some studies additionally highlighted cognitive difficulties, and persistent interictal symptoms. Based on these definitions, seven overarching conceptual domains were identified: functional impairment, social and relational impact, psychological and emotional distress (including anticipatory anxiety and fear of the next attack as a specific subtheme primarily informed by the FAMI and Eurolight studies), cognitive dysfunction, sensory symptoms, physical symptoms, and behavioral modifications (Fig. 2).

The study-level domain mapping is presented in Supplementary Table 2. This table distinguishes explicit study-level reporting (✓) from instrument item-level coverage (○). When considering explicit reporting alone, functional impairment was the most frequently discussed domain (17 studies, 59%), followed by psychological/emotional distress (11 studies, 38%) and social/relational impact (10 studies, 34%), whereas cognitive dysfunction (3 studies, 10%), sensory symptoms (2 studies, 7%), and physical symptoms (1 study, 3%) were rarely addressed. Behavioral modifications were explicitly discussed in 5 studies (17%). When instrument item-level coverage was included, the four domains captured by the MIBS-4 reached near-complete coverage (functional impairment 93%, social/relational impact 90%, psychological/emotional distress 100%, behavioral modifications 100%), while the three domains not captured by the MIBS-4 remained rarely addressed (cognitive dysfunction 10%, sensory symptoms 7%, physical symptoms 3%).

Of these seven domains, the MIBS-4 explicitly captures four—functional impairment, social/relational impact, planning difficulty (as a proxy for behavioral modification), and emotional/cognitive distress—while sensory and physical symptoms, broader cognitive deficits, and anticipatory fear are not directly assessed. In contrast, the FAMI primarily focuses on anticipatory fear and emotional distress, addressing other domains insofar as they relate to these processes. A notable conceptual extension identified in the literature is the notion of cumulative life-course burden, as described in the Eurolight project, which emphasizes that interictal burden is not merely a transient state between attacks but accumulates over time, influencing life decisions, career trajectories, and social development.

### Measurement properties

The distribution of evidence on measurement properties was markedly uneven across the nine COSMIN domains. Construct validity (hypothesis testing) was by far the most frequently evaluated property (19 studies), followed by responsiveness (8 studies). In contrast, structural validity was assessed in two studies, internal consistency in three, and test-retest reliability in one. Notably, measurement error and criterion validity were not assessed in any of the included studies.

#### Internal consistency/reliability

Two studies demonstrated good internal consistency of the MIBS-4, with Cronbach’s alpha values ranging from 0.83 to 0.85 in the Turkish and Portuguese versions, respectively [23, 24]. Internal consistency of the FAMI was also high, with McDonald’s ω values indicating good to excellent reliability across subscales (0.85–0.91) [22]. Test–retest reliability was evaluated for the Turkish Version of the MIBS-4, showing good to near-excellent reliability (ICC = 0.898) [24].

#### Content validity

One study addressed the content validity of the MIBS-4. Buse et al. (2007) described the initial development of the four-item scale at the American Academy of Neurology conference, reporting that items were generated to capture four core domains of interictal burden: work/school impairment, family/social disruption, difficulty making plans, and emotional/cognitive distress (21). However, this report was published solely as a conference abstract; no peer-reviewed full paper describing the item generation process, qualitative methods, patient involvement, or expert review has been identified. Content validity for both the Eurolight Questionnaire and the FAMI was not explicitly assessed in any of the included studies.

#### Structural validity

One study provided evidence for the structural validity of the MIBS-4. Malheiro et al. (2025) reported in principal component analysis strong factor loadings for all four MIBS-4 items (0.706–0.785), supporting good structural validity of the Portuguese MIBS-4 [23]. Structural validity was also demonstrated for the FAMI, with exploratory factor analysis identifying three clearly interpretable factors and confirmatory factor analysis indicating an acceptable to good model fit.

#### Construct validity

Construct validity, assessed through hypothesis testing, was the most extensively evaluated measurement property, addressed in 19 studies. These studies consistently demonstrated associations between interictal burden scores and several PROMs that measure related, but distinct constructs. An overview of construct validity findings is presented in Table 2.

#### Convergent validity

Moderate correlations have been observed between the MIBS-4 and general quality-of-life measures such as WHO-5 Well-Being Index [25], 12-Item Short Form Health Survey [23], and Migraine-Specific Quality of Life Questionnaire (MSQ) [26]. Moderate associations were also found with measures of headache-related disability, including MIDAS [23, 26] and HIT-6 [27, 28]. A graded association between MIBS-4 and both MIDAS and MSQ was also reported in the OVERCOME Japan survey [29, 30]. Similarly, migraine-related stigma, assessed using the Migraine-Related Stigma Scale (MiRS), demonstrated a graded association with MIBS-4 in both the OVERCOME Japan and US surveys [31, 32]. Also, general anxiety, as measured by the Generalized Anxiety Disorder 7-item scale (GAD-7), and depression, assessed using the Patient Health Questionnaire (PHQ), showed moderate correlations with MIBS-4 [26]. Likewise, both the HeAD-US study and a smaller cross-sectional observational study demonstrated that anxiety and depressive symptoms were significantly associated with higher MIBS-4 scores [33, 34]. Findings for migraine frequency were mixed, with correlations ranging from strong [16], to moderate [7], and weak [26] correlations with MIBS-4.

For the FAMI, convergent validity was supported by moderate correlations with the HIT-6 (ρ = 0.50) and the GAD-7 (ρ = 0.54), alongside a weak correlation with migraine days (ρ = 0.19) [22]. Additional cross-sectional evidence confirmed construct validity through an observed association between FAMI and HIT-6 [19].

One study utilized interictal burden items from the Eurolight questionnaire [20], providing evidence of construct validity through demonstrated associations between interictal symptoms and headache frequency, intensity, and productivity loss. 

#### Known-groups validity

Population-based studies demonstrate that the MIBS-4 can discriminate between clinically relevant subgroups. In the OVERCOME EU survey (*n* = 20,756), Evers et al. (2024) reported a graded association between headache frequency and severe interictal burden (MIBS-4 ≥ 5), with the proportion increasing from 41.2% among those with 0–3 headache days/month to 57.8% among those with ≥ 15 headache days/month [34]. Similarly, data from the OVERCOME Japan survey showed higher MIBS-4 scores in individuals with menstrual migraine compared with non-menstrual migraine [35].

**Table 2:** Construct validity across studies

Instrument	Author (Year)	Type of construct validity	Comparator variable	Result
**MIBS-4**	Argyriou et al. (2024)	Convergent	Headache frequency	*r* = 0.68
	Malheiro et al. (2025)	Convergent	SF-12	*r* = 0.48
			MIDAS	*r* = 0.30
			HADS	*r* = 0.21
	Lampl et al. (2024)	Convergent	WHO-5	*r* = − 0.36
	Sandoe et al. (2021)	Convergent	MSQ	*r* = − 0.53
			MIDAS	*r* = 0.41
			Migraine frequency	*r* = 0.22
			PHQ-9	*r* = 0.55
			GAD-7	*r* = 0.42
			PGI-S	*r* = 0.32
	Hubig et al. (2022)	Convergent	Migraine frequency	*r* = 0.40
			Headache frequency	*r* = 0.35
			HIT-6	*r* = 0.37
	Hashimoto et al. (2025)	Convergent	HIT-6	*r* ≈ 0.56 (derived from R² = 0.309; *p* < 0.0001)
	Karaci et al.	Convergent	HIT-6	ρ = 0.33
			MIDAS	ρ = 0.29
	Takizawa et al. (2025)	Convergent	MIDASMSQ	Graded association
	Shapiro et al. (2024)	Convergent	MiRS	Graded association
	Igarashi et al. (2024)	Convergent	MiRS	Graded association
	Sarkar et al. (2025)	Convergent	PHQ-4	Anxiety/depression associated with 11.7%/15.5% higher MIBS scores (all *p* < 0.05).
	Sotero et al. (2025)	Convergent	HIT-6SMCHADS	Higher burden (MIBS-4 ≥3) associated with increased headache frequency, intensity, HIT-6, memory complaints anxiety, and depression (all *p* ≤ 0.002)
	Awaki et al. (2024)	Convergent	MSQ	Significant association with MIBS-4 burden
	Pascual et al. (2023)	Convergent	Headache frequency	Higher burden associated with ≥ 15 headache days
	Evers et al. (2024)	Known-groups	Headache frequency categories	Graded association across headache frequency categories
	Takizawa et al. (2025)	Known-groups	MM vs. Non-MM	Significant higher MIBS-4 scores in MM vs. Non-MM (3.7 vs. 3.1; *p* < 0.001)
**Eurolight Questionnaire**	Lampl et al. (2016)	Convergent	Headache intensity Headache frequencyLost productive time	Graded association
**FAMI**	Klan et al. (2022)	Convergent	HIT-6	ρ = 0.50
			GAD-7	ρ = 0.54
			Migraine frequency	ρ = 0.19
	Kaske et al. (2025)	Convergent	HIT-6	Graded association

SF-12, 12-Item Short Form Health Survey; MIDAS, Migraine Disability Assessment; PHQ-9, 9-Item Patient Health Questionnaire; WHO-5, World Health Organization–5 Well-Being Index; MSQ, Migraine-Specific Quality of Life Questionnaire; HIT-6, 6-Item Headache Impact Test; MiRS, Migraine Interictal Burden Scale; PHQ-4, 4-Item Patient Health Questionnaire; SMC, Subjective Memory Complaints; HADS, Hospital Anxiety and Depression Scale; GAD-7, 7-Item Generalized Anxiety Disorder Scale; MM, Menstrual Migraine; Non-MM, Non-Menstrual Migraine; r, Pearson correlation coefficient; ρ (rho), Spearman rank correlation coefficient

#### Cross-cultural validity

Two studies addressed cross-cultural validity through formal linguistic adaptation, evaluating the Portuguese and Turkish versions of the MIBS-4, both of which demonstrated adequate structural validity, internal consistency, and test–retest reliability, as reported above [23, 24]. No other language adaptations with formal psychometric evaluation, nor any language adaptations were identified for the FAMI and the Eurolight questionnaire.

#### Responsiveness

Eight studies provided evidence on the responsiveness of the MIBS-4. Two studies reported significant reductions in MIBS-4 scores following treatment with onabotulinumtoxinA [16, 17]. In addition, both open-label studies and randomized controlled trials demonstrated significant reductions in MIBS-4 scores with calcitonin gene-related peptide (CGRP)-targeted therapies, including eptinezumab [36, 37], galcanezumab [38] atogepant [39] and rimegepant [18]. Only one study reported no significant change after 3 months of atogepant treatment [40]. The overall responsiveness of the MIBS-4 across preventive therapies is systematically summarized in Table 3. For FAMI and Eurolight, responsiveness has not been evaluated in the included studies.

**Table 3:** Responsiveness of MIBS-4 across studies

Author (Year)	Study Design	*N*	Age (mean)	Female (%)	Migraine Type	Treatment	Outcome
Argyriou et al. (2024)	Prospective observational (open-label) study	70	44.4	91.5	CM	OnabotulinumtoxinA	Median MIBS-4 significantly reduced after 3 treatment cycles (− 7; *p* < 0.001)
Barbanti et al. (2025a)	Prospective observational (real-world) study	74	47.9	73	EM + CM	Eptinezumab	Significant reduction at 24 weeks (− 4.3; *p* < 0.001)
Barbanti et al. (2025b)	Prospective observational (real-world) study	82	45.3	91.5	EM + CM	Atogepant	Significant reduction at 12 weeks (− 5.4; *p* < 0.001)
Barbanti et al. (2024)	Prospective observational (real-world) study	26	49.7	81.8	EM + CM	Eptinezumab	Significant reduction at 12 weeks (− 4.6; *p* < 0.001)
Lipton et al. (2023)	Randomized controlled trial	462	45.7	85.9	EM + CM	Galcanezumab	Greater reduction vs. placebo at 3 months (− 1.1; *p* < 0.001)
Sánchez-Huertas et al. (2025)	Prospective observational study	137	44	91.3	CM	OnabotulinumtoxinA	Mean MIBS-4 decreased from 8.47 at baseline to 5.97 at 3 months and 4.86 at 12 months (*p* < 0.001); median 9 → 6 → 5
Vernieri et al. (2025)	Prospective observational (real-world) study	106	50.6	87.7	EM + CM	Atogepant	No significant change at 3 months (*p* = 0.237)
Pozo-Rosich et al. (2025)	Randomized controlled trial	652	42.3	86	EM	Rimegepant	Greater reduction vs. placebo at 3 months (− 0.9; *p* < 0.001)

EM, episodic migraine; CM, chronic migraine

### Methodological quality

The COSMIN Risk of Bias checklist was applied to all study–property combinations across 29 assessable studies (see Supplementary Table 3). Among studies evaluating the MIBS-4, construct validity was most frequently assessed, with methodological quality ranging from adequate to doubtful. Responsiveness was generally rated as adequate, with several studies achieving very good ratings, particularly those employing randomized controlled designs with minimal methodological limitations [18, 38]. Evidence for structural validity and internal consistency of the original English version of the MIBS-4 was limited; however, studies of translated versions (Turkish and Portuguese) demonstrated adequate to very good methodological quality [23, 24]. Reliability (test–retest) of the MIBS-4 was rated as doubtful, primarily due to insufficient information regarding the stability of patients between assessments [24]. Content validity and cross-cultural validity were rarely evaluated and, when assessed, were rated as inadequate, because appropriate methods (e.g., measurement invariance or Differential Item Functioning analyses) were not applied. Notably, no studies reported on measurement error or criterion validity for the MIBS-4. All three conference abstracts received “Inadequate” ratings due to insufficient methodological reporting, including the original MIBS-4 development paper [21].

One study evaluated the Eurolight questionnaire, in which construct validity was rated as inadequate, as no information was provided regarding the measurement properties of the comparator instruments [20]. For the FAMI, structural validity and internal consistency were each assessed in one study and were both rated as very good, as confirmatory factor analysis was appropriately performed [22]. Construct validity, evaluated in two studies, demonstrated mixed methodological quality, with one study rated as adequate and the other as doubtful.

### Quality of evidence

The COSMIN-modified GRADE approach was applied to synthesize the quality of evidence for each instrument across all nine measurement properties (Table 4). For the MIBS-4, most measurement properties demonstrated sufficient results, supported by moderate to high quality of evidence. Internal consistency demonstrated sufficient results with high-quality evidence and no downgrading, with Cronbach’s alpha ≥ 0.70 reported in two studies [23, 24]. Structural validity and reliability also demonstrated sufficient results, supported by moderate-quality evidence, with evidence of good model fit and intraclass correlation coefficients (ICC) ≥ 0.70 [24]. Construct validity and responsiveness were also supported by moderate-quality evidence, with at least 75% of predefined hypotheses confirmed for related constructs and changes in MIBS scores. In contrast, content validity, measurement error, and criterion validity were either insufficiently assessed or not reported, resulting in indeterminate ratings. Cross-cultural validity was rated as indeterminate with very low-quality of evidence, due to the use of inappropriate methods.

For the Eurolight questionnaire, construct validity was assessed in a single study and showed a sufficient result with low-quality evidence, downgraded due to lack of information on the measurement properties of comparator instruments. For the FAMI, structural validity and internal consistency were assessed in one study each and showed sufficient results of high-quality evidence. Construct validity, assessed in two studies, also demonstrated sufficient results with moderate-quality evidence, downgraded due to suboptimal statistical methods.

Based on the COSMIN recommendation framework, the MIBS-4 was classified as Category B, indicating that it has potential for use but requires further validation. The Eurolight questionnaire and FAMI were not assigned a positive recommendation for standalone interictal burden measurement because the available evidence was limited.

**Table 4:** Summary of findings (COSMIN-modified GRADE)

Instrument	Property	Studies (*n*)	*N*	Result	Quality of Evidence	Downgrading reasons
MIBS-4	Content validity	1	unknown	Indeterminate (?)	--	
	Structural validity	1	459	Sufficient (+)	Moderate	Risk of bias: Exploratory factor analysis instead of confirmatory factor analysis performed (-1)
	Internal consistency	2	637	Sufficient (+)	High	No downgrading
	Reliability (test-retest)	1	178	Sufficient (+)	Moderate	Risk of bias: Unclear if patients were stable (-1)
	Measurement error	0	0	Indeterminate (?)	--	
	Criterion validity	0	0	Indeterminate (?)	--	
	Construct validity	16	> 100,000	Sufficient (+)	Moderate	Risk of bias: statistical method applied not optimal (-1)
	Cross-cultural validity	2	1609	Indeterminate (?)	Very low	Inappropriate methods: no invariance/DIF testing (− 3)
	Responsiveness	8	1781	Sufficient (+)	Moderate	Risk of bias: statistical method applied not optimal (-1)
Eurolight Questionnaire	Content validity	0	0	Indeterminate (?)	--	
	Structural validity	0	0	Indeterminate (?)	--	
	Internal consistency	0	0	Indeterminate (?)	--	
	Reliability	0	0	Indeterminate (?)	--	
	Measurement error	0	0	Indeterminate (?)	--	
	Criterion validity	0	0	Indeterminate (?)	--	
	Construct validity	1	6455	Sufficient (+)	Low	Risk of bias: No information on the measurement properties of the comparator instruments (-2)
	Cross-cultural validity	0	0	Indeterminate (?)	--	
	Responsiveness	0	0	Indeterminate (?)	--	
FAMI	Content validity	0	0	Indeterminate (?)	--	
	Structural validity	1	387	Sufficient (+)	High	No downgrading
	Internal consistency	1	387	Sufficient (+)	High	No downgrading
	Reliability	0	0	Indeterminate (?)	--	
	Measurement error	0	0	Indeterminate (?)	--	
	Criterion validity	0	0	Indeterminate (?)	--	
	Construct validity	2	433	Sufficient (+)	Moderate	Risk of bias: statistical method applied not optimal (-1)
	Cross-cultural validity	0	0	Indeterminate (?)	--	
	Responsiveness	0	0	Indeterminate (?)	--	

## Discussions

This systematic review is, to our knowledge, the first to apply the COSMIN methodology to evaluate the measurement properties of patient-reported outcome measures assessing interictal burden in migraine. From 29 included studies published between 2007 and 2025, we identified three instruments: The Migraine Interictal Burden Scale (MIBS-4), the Eurolight questionnaire, and the Fear of Attacks in Migraine Inventory (FAMI). The MIBS-4 dominated the literature, whereas the Eurolight questionnaire and FAMI were each evaluated in only one or two studies, respectively. Notably, 69% of included studies were published in 2024 or later, indicating that interictal burden measurement is a rapidly evolving field, yet one that has proceeded largely without the foundational psychometric work recommended by contemporary standards.

The evidence base of measurement properties was heavily skewed toward construct validity (19 studies) and responsiveness (8 studies), with critical content validity, structural validity, test–retest reliability, and measurement error either absent or supported by only one or two studies each. The most striking finding of this review is that the MIBS-4, although being by far the most widely adopted measure of interictal burden in migraine, lacks peer-reviewed evidence for fundamental measurement properties that COSMIN guidelines consider essential for recommending a PROM. The sole content validity evidence derives from a 2007 conference abstract presented at the American Academy of Neurology [21], with no subsequent peer-reviewed publication detailing the item generation process, patient involvement in development, or content validity assessment. Structural validity has been examined in only one study, but none employed confirmatory factor analysis, item response theory, or Rasch modeling—the analytic approaches recommended by COSMIN. Measurement error indices such as the standard error of measurement, smallest detectable change, and limits of agreement have never been reported. Consequently, despite demonstrating sufficient construct validity and responsiveness, the MIBS-4 remained classified as Category B because, under the COSMIN recommendation framework, missing evidence for essential measurement properties (i.e. structural validity and measurement error) automatically caps the highest possible recommendation at Category B. Similar gaps were identified for the FAMI and Eurolight questionnaire, although these instruments have been studied far less extensively.

These findings likely reflect a broader phenomenon in headache research wherein instruments gain clinical traction through repeated use in treatment trials before their psychometric foundations are rigorously established. The MIBS-4’s brevity (four items), face validity, and free availability have facilitated its rapid adoption, particularly in clinical trials of CGRP-targeted therapies and onabotulinumtoxinA. The evidence for construct validity is encouraging, as moderate correlations with PHQ-9, MSQ, HIT-6 and MIDAS align with theoretical expectations that interictal burden is associated with, but not fully explained by, attack-related disability, overall well-being, and functional impairment. However, construct validity alone cannot compensate for the absence of evidence regarding whether the instrument measures a coherent unidimensional construct (structural validity), whether scores are reproducible over time in stable patients (reliability), or what magnitude of change constitutes measurement noise versus true change (measurement error). Conversely, the absence of criterion validity across all instruments is expected, as no gold standard for measuring interictal burden currently exists.

Notably, one study reported no significant change in MIBS-4 scores despite significant reductions in migraine frequency and disability with atogepant [40], whereas another study using the same treatment demonstrated significant MIBS-4 improvement [39], This discrepancy likely reflects differences in baseline disease severity and prior treatment failure, but also raises the possibility that interictal burden may not respond uniformly to preventive treatment, particularly when baseline interictal burden is moderate rather than severe. This observation underscores the importance of establishing a minimal important change threshold for the MIBS-4 to distinguish clinically meaningful improvement from statistical noise.

Our synthesis of 21 definitions of interictal burden across the included literature yielded seven conceptual domains: functional impairment, social and relational impact, psychological and emotional distress, cognitive dysfunction, sensory symptoms, physical symptoms, and behavioral modifications. The MIBS-4, a four-item instrument, captures approximately four of these domains, leaving sensory symptoms, physical symptoms, and anticipatory fear of attacks inadequately represented. Conversely, the FAMI primarily captures anticipatory fear and emotional distress, while the Eurolight project highlights interictal burden as a cumulative life-course impact rather than a transient state between attacks.

This review complements and extends several prior syntheses. Vincent et al. (2022) provided a narrative overview of interictal burden concepts but did not systematically evaluate measurement properties [9]. Braganza et al. (2022) conducted a meta-analysis of interictal cognitive deficits, confirming the cognitive domain we identified but focusing on neuropsychological test performance rather than PROMs [8]. Lo et al. (2022) used qualitative interviews to explore patient experiences of interictal burden, identifying themes—including unpredictability, identity disruption, and social withdrawal—that align closely with our seven-domain framework and reinforce the argument that current instruments capture only a subset of the lived experience [10]. Brandes (2008) presciently described the full burden within the migraine cycle, and it is notable that nearly two decades later, the measurement infrastructure for the interictal phase remains underdeveloped relative to the ictal phase [6].

This review has several strengths. We applied the COSMIN methodology prospectively, including its risk-of-bias checklist and criteria for good measurement properties, providing a standardized and reproducible evaluation framework. The systematic search across multiple databases was comprehensive, and the inclusion of conference abstracts ensured capture of the earliest MIBS-4 development work. The synthesis of conceptual domains from 23 definitions provides a framework that can inform future instrument development. Registration and adherence to PRISMA guidelines enhance transparency.

Several limitations should be considered. First, identifying overlapping cohorts, particularly within the OVERCOME program and the CONQUER trial, required interpretive judgment. Second, the predominance of the MIBS-4 means that our findings largely reflect the evidence base for a single instrument; the Eurolight questionnaire and FAMI have been evaluated too infrequently to allow robust conclusions, although findings for the FAMI are promising. Third, the rapidly expanding literature raises the possibility that additional relevant evidence may have emerged during the review process. Fourth, we did not perform a meta-analysis, in line with COSMIN recommendations for systematic reviews of measurement properties, which limits the ability to provide pooled estimates. Finally, the absence of a peer-reviewed development paper for the MIBS-4 constrained a comprehensive assessment of content validity, as the available conference abstract provides only limited methodological detail. Relatedly, the OVERCOME survey program contributed a substantial proportion of the evidence base, with analyses from the Japan first and second waves, the United States, the European Union, and Spain [41, 42]. Although each wave recruited independent participants and addressed distinct research questions, the shared survey infrastructure, common instruments, and overlapping investigator teams may limit the independence of the evidence. This concentration also means that the construct validity evidence is disproportionately drawn from web-based population surveys, which may not generalize to clinical populations.

For clinicians, our findings suggest that the MIBS-4 can be used as a practical screening tool for interictal burden, supported by reasonable construct validity evidence and demonstrated sensitivity to treatment effects. However, clinicians should be cautious about interpreting small changes in MIBS-4 scores as clinically meaningful, given the absence of measurement error data and formally derived minimal important change thresholds. Categorical scoring (no, mild, moderate, severe burden) may be more interpretable than total scores for clinical decision-making until more robust psychometric data become available. Clinicians should also recognize that a low MIBS-4 score does not necessarily indicate low interictal burden, as domains such as sensory sensitivity, physical symptoms, and anticipatory anxiety are not captured by this instrument.

Future research priorities are clear. First, a comprehensive psychometric evaluation of the MIBS-4 addressing all COSMIN measurement properties in a single, adequately powered study is urgently needed. This should include a peer-reviewed content validity study, incorporating cognitive interviews with patients and expert input, as well as structural validity analyses using confirmatory factor analysis or item response theory in large, diverse samples. In addition, test–retest reliability and measurement error should be established in stable populations, and anchor-based methods should be applied to determine the minimal important change, thereby improving interpretability in clinical trials. Second, the development of a new or expanded instrument, grounded in the seven-domain conceptual framework and supported by robust patient involvement in item generation, may address the content gaps identified in this review. Third, formal cross-cultural validation studies are required for the numerous language versions currently in use (e.g., Japanese, Spanish, German), as simple translation alone is insufficient. Finally, future research should evaluate whether interictal burden measures can inform treatment selection or predict treatment response, thereby establishing clinical utility beyond outcome assessment. In addition, future studies should elucidate in how far interictal burden is proportionally related to the number and severity of attacks, or whether the interictal burden represents an independent entity with its own pathophysiological mechanisms.

Our findings raise a practical question: should future efforts focus on refining the MIBS-4 or on developing a new instrument? We suggest that these are complementary rather than competing priorities. The MIBS-4 fills an important niche as a brief, four-item screening tool that can be feasibly integrated into routine clinical encounters and large-scale clinical trials. Its widespread adoption across multiple countries and therapeutic contexts represents a substantial practical advantage that should not be discarded. However, its four items cannot be expected to capture all seven identified domains, and the absence of foundational psychometric evidence (content validity, structural validity, measurement error) means that critical questions about what the MIBS-4 actually measures remain unanswered. We therefore recommend a dual strategy: (1) conducting the missing psychometric studies for the MIBS-4 to determine whether it functions as intended as a brief screener, and (2) developing a more comprehensive instrument informed by the seven-domain framework identified in this review, with robust patient involvement in item generation as recommended by COSMIN. A modular approach — retaining the MIBS-4 for screening while adding domain-specific modules for research or specialized clinical contexts — may balance comprehensiveness with respondent burden.

## Conclusion

This COSMIN systematic review reveals a striking disparity between the widespread adoption of the MIBS-4 as the predominant measure of interictal burden in migraine and the limited psychometric evidence supporting its use. While construct validity and sensitivity to change are reasonably well documented across 19 and 8 studies respectively, fundamental measurement properties—content validity, structural validity, reliability, and measurement error—remain inadequately evaluated. The interictal burden construct encompasses at least seven domains, of which the MIBS-4 captures only a subset. This review cannot yet fully recommend the MIBS-4 as a sufficiently validated instrument by COSMIN standards, though its practical utility as a brief clinical screening tool is acknowledged. Rigorous psychometric studies addressing the identified evidence gaps, alongside conceptual work toward more comprehensive assessment of interictal burden, are essential to ensure that this rapidly growing field is built on a sound measurement foundation.

## Supplementary Information

Below is the link to the electronic supplementary material.

Supplementary Material 1

## Data Availability

No datasets were generated or analysed during the current study.

## Declarations

### Author information

This work was conducted within the framework of the European Headache Federation School of Advanced Studies (EHF-SAS) consortium, comprising the following members: Cornelius Angerhöfer (cornelius.angerhoefer@charite.de), Yutaro Fuse (yfuse@chiba-u.jp) Tom Canning (Thomas.2.canning@kcl.ac.uk), Alice C. von Alvensleben (alice.vonalvensleben@charite.de), Sheharyar Ahmad (sheharyar.ahmad@unicampania.it) Samuel van Langenhove (vanlangenhovesamuel@gmail.com), Ane Murillo (ane.murillo@vallhebron.cat), Laia Boada (laia.boada@vallhebron.cat), Maria Roque (maria0roque@gmail.com), Antoinette MaassenVanDenBrink (a.vanharen-maassenvandenbrink@erasmusmc.nl) and Christian Lampl (dr-lampl@a1.net).

## Abbreviations

CGRP: Calcitonin Gene-Related Peptide
CM: Chronic MIGRAINE
COSMIN: Consensus-based Standards for the Selection of Health Measurement Instruments
EM: Episodic Migraine
FAMI: Fear of Attacks in Migraine Inventory
GAD-7: Generalized Anxiety Disorder 7-item Scale
GRADE: Grading of Recommendations Assessment, Development and Evaluation
HeAD-US: Headache, Anxiety and Depression Study – United States
HIT-6: Headache Impact Test-6
ICC: Intraclass Correlation Coefficient
ICHD-3: International Classification of Headache Disorders, 3rd Edition
MIBS-4: Migraine Interictal Burden Scale-4
MIDAS: Migraine Disability Assessment
MiRS: Migraine-Related Stigma Scale
MSQ: Migraine-Specific Quality of Life Questionnaire
PHQ: Patient Health Questionnaire
PRISMA: Preferred Reporting Items for Systematic Reviews and Meta-Analyses
PROMs: Patient-Reported Outcome Measures
PROSPERO: International Prospective Register of Systematic Reviews
SF-12: 12-Item Short Form Health Survey
SF-36: 36-Item Short Form Health Survey
WHO-5: World Health Organization-Five Well-Being Index
WPAI: Work Productivity and Activity Impairment Questionnaire
