# Supplementary material for: Interictal burden in migraine: a systematic review of conceptual domains and measurement properties of instruments
Source: J Headache Pain. 2026 Jul 23;27(1):190. doi: 10.1186/s10194-026-02458-0 (PMC13401311; doi:10.1186/s10194-026-02458-0)
Supplement: Supplementary file 1 — Supplementary Material 1 [file 10194_2026_2458_MOESM1_ESM.docx]

# **Supplement material: Interictal burden in migraine: a systematic review of conceptual domains and measurement properties of instruments**

**Table 1. Definitions of interictal burden reported across studies that provided an explicit definition**

| **Study** | **Definition/Description of Interictal Burden** |
| --- | --- |
| Argyriou et al. 2024 | Burden of disease between migraine attacks on the domains of patients' quality of life, including productivity, cognitive ability and psychosocial as well as emotional well-being |
| Awaki et al. 2024 | Impairment between migraine attacks |
| Barbanti et al. 2025a | Burden of migraine during headache-free periods, measured with the MIBS-4 |
| Buse et al. 2007 | The burden of migraine between attacks including impairment in work/school, family/social life, difficulty making plans, emotional/cognitive distress |
| Evers et al. 2024 | Interictal burden and unpredictability of attacks can result in significant disruption to daily life |
| Garcia-Azorin et al. 2020 | The disruptive effects of migraine occur both during attacks (ictal) and between attacks (interictal), affecting work, school, family, and social life |
| Kaske et al. 2025 | Fear of attacks as component of interictal burden; anticipatory anxiety about next migraine attack |
| Hashimoto et al. 2025 | Interictal disability including difficulties in maintaining work productivity, social participation, or family responsibilities |
| Hubig et al. 2022 | The impact of migraine on migraine-free days (interictal) |
| Igarashi et al. 2023 | Higher levels of burden between migraines |
| Lampl et al. 2016 | Symptoms between headache attacks including anxiety, avoidance behavior, and cumulative life-course burden (education, career, earnings) |
| Lampl et al. 2024 | The total burden of migraine includes not only the episodes with headache pain but extends throughout the interictal periods |
| Lipton et al. 2023 | The disruptive effects of migraine occur both during attacks (ictal) and between attacks (interictal), affecting work, school, family, and social life |
| Malheiro et al. 2025 | Impairment between migraine episodes, frequently overlooked compared to attack frequency and intensity |
| Matsumori et al. 2022 | Burden related to migraine in the time between attacks, including disruption at work and school, diminished family and social life, difficulty planning, and emotional difficulty |
| Pascual et al. 2023 | Burden related to headache between attacks, measured by the MIBS-4 |
| Sánchez-Huertas et al. 2025 | Symptoms and impairments between migraine attacks, including cognitive dysfunction, photophobia, and fatigue |
| Sarkar et al. 2025 | Impact on both disability during and between attacks, assessed by MIBS |
| Shapiro et al. 2024a | Burden of migraine between attacks, assessed on days without migraine across four domains: impairment in work/school, social/leisure planning, life impact on headache-free days, and emotional/cognitive distress |
| Sotero et al. 2025 | The interictal burden of migraine encompasses the overall impact of the disease between migraine episodes |
| Takizawa et al. 2025a | Burdens between headaches (interictal period) negatively affect multiple aspects of daily life independently of the impact of headaches |

**Table 2. Study-level mapping of interictal burden conceptual domain coverage**

**Study-level mapping of interictal burden conceptual domain coverage across 29 included studies.**

| **Study** | **Year** | **Instrument** | **Functional impairment** | **Social and relational impact** | **Psychological and emotional distress** | **Cognitive dysfunction** | **Sensory symptoms** | **Physical symptoms** | **Behavioral modifications** |
| --- | --- | --- | --- | --- | --- | --- | --- | --- | --- |
| Argyriou et al. | 2024 | MIBS-4 | ✓ | ✓ | ✓ | ✓ | ✓ |  | ○ |
| Awaki et al. | 2024 | MIBS-4 | ✓ | ✓ | ○ |  |  |  | ○ |
| Barbanti et al. | 2024 | MIBS-4 | ○ | ○ | ○ |  |  |  | ○ |
| Barbanti et al. | 2025a | MIBS-4 | ○ | ○ | ○ |  |  |  | ○ |
| Barbanti et al. | 2025b | MIBS-4 | ○ | ○ | ○ |  |  |  | ○ |
| Buse et al. | 2007 | MIBS-4 | ✓ | ✓ | ✓ |  |  |  | ✓ |
| Evers et al. | 2024 | MIBS-4 | ✓ | ○ | ○ |  |  |  | ○ |
| García-Azorín et al. | 2020 | MIBS-4 | ✓ | ✓ | ○ |  |  |  | ○ |
| Hashimoto et al. | 2025 | MIBS-4 | ✓ | ✓ | ○ |  |  |  | ○ |
| Hubig et al. | 2022 | MIBS-4 | ✓ | ○ | ○ |  |  |  | ○ |
| Igarashi et al. | 2024 | MIBS-4 | ○ | ✓ | ✓ |  |  |  | ○ |
| Karaci et al. | 2024 | MIBS-4 | ○ | ○ | ○ |  |  |  | ○ |
| Kaske et al. | 2025 | FAMI |  |  | ✓ |  |  |  | ○ |
| Klan et al. | 2022 | FAMI |  |  | ✓ |  |  |  | ✓ |
| Lampl et al. | 2016 | Eurolight | ✓ |  | ✓ |  |  |  | ✓ |
| Lampl et al. | 2024 | MIBS-4 | ✓ | ○ | ✓ |  |  |  | ○ |
| Lipton et al. | 2023 | MIBS-4 | ✓ | ✓ | ○ |  |  |  | ○ |
| Malheiro et al. | 2025 | MIBS-4 | ✓ | ○ | ○ |  |  |  | ○ |
| Matsumori et al. | 2022 | MIBS-4 | ✓ | ✓ | ✓ |  |  |  | ✓ |
| Pascual et al. | 2023 | MIBS-4 | ✓ | ○ | ○ |  |  |  | ○ |
| Pozo-Rosich et al. | 2025 | MIBS-4 | ○ | ○ | ○ |  |  |  | ○ |
| Sánchez-Huertas et al. | 2025 | MIBS-4 | ○ | ○ | ○ | ✓ | ✓ | ✓ | ○ |
| Sandoe et al. | 2021 | MIBS-4 | ○ | ○ | ✓ |  |  |  | ○ |
| Sarkar et al. | 2025 | MIBS-4 | ✓ | ○ | ✓ |  |  |  | ○ |
| Shapiro et al. | 2024 | MIBS-4 | ✓ | ✓ | ✓ |  |  |  | ✓ |
| Sotero et al. | 2025 | MIBS-4 | ✓ | ○ | ○ | ✓ |  |  | ○ |
| Takizawa et al. | 2025a | MIBS-4 | ✓ | ✓ | ○ |  |  |  | ○ |
| Takizawa et al. | 2025b | MIBS-4 | ○ | ○ | ○ |  |  |  | ○ |
| Vernieri et al. | 2025 | MIBS-4 | ○ | ○ | ○ |  |  |  | ○ |
| **Explicit (✓)** |  |  | **17 (59%)** | **10 (34%)** | **11 (38%)** | **3 (10%)** | **2 (7%)** | **1 (3%)** | **5 (17%)** |
| **Item coverage (○)** |  |  | **10 (34%)** | **16 (55%)** | **18 (62%)** | **0 (0%)** | **0 (0%)** | **0 (0%)** | **24 (83%)** |
| **Total (✓+○)** |  |  | **27 (93%)** | **26 (90%)** | **29 (100%)** | **3 (10%)** | **2 (7%)** | **1 (3%)** | **29 (100%)** |

*A check mark (✓) indicates that the study authors explicitly defined, discussed, or analysed the domain as a component of interictal burden in their text. An open circle (○) indicates that the domain is covered by the item content of the instrument used (e.g., the four MIBS-4 items correspond to functional impairment, social/relational impact, psychological/emotional distress, and behavioral modifications; the FAMI corresponds to psychological/emotional distress and behavioral modifications) but was not explicitly discussed by the study authors as an individual domain. The cognitive dysfunction domain was coded only when a study explicitly discussed cognitive ability, concentration, memory, or cognitive processing; the broad "emotional and cognitive distress" item on the MIBS-4 was mapped to psychological/emotional distress rather than cognitive dysfunction. The Instrument column identifies the primary interictal burden instrument used in each study. Coverage counts are provided separately for explicit reporting (✓), instrument item coverage (○), and combined total (✓ + ○).* *MIBS-4, Migraine Interictal Burden Scale-4; FAMI, Fear of Attacks in Migraine Inventory; Eurolight, Eurolight interictal burden questionnaire.*

**Table 3. Risk of Bias Assessment**

| **Author** | **Year** | **Instrument** | **Content_Validity** | **Structural_Validity** | **Internal_Consistency** | **Reliability** | **Measurement_Error** | **Criterion_Validity** | **Construct_Validity** | **Cross_Cultural_Validity** | **Responsiveness** |
| --- | --- | --- | --- | --- | --- | --- | --- | --- | --- | --- | --- |
| Argyriou et al. | 2024 | MIBS-4 |  |  |  |  |  |  | Adequate |  | Adequate |
| Awaki et al. | 2024 | MIBS-4 |  |  |  |  |  |  | Doubtful |  |  |
| Barbanti et al. | 2024 | MIBS-4 |  |  |  |  |  |  |  |  | Adequate |
| Barbanti et al. | 2025a | MIBS-4 |  |  |  |  |  |  |  |  | Adequate |
| Barbanti et al. | 2025b | MIBS-4 |  |  |  |  |  |  |  |  | Adequate |
| Buse et al. | 2007 | MIBS-4 | Inadequate | Inadequate |  |  |  |  |  | Inadequate |  |
| Evers et al. | 2024 | MIBS-4 |  |  |  |  |  |  | Doubtful |  |  |
| Garcia-Azorin et al. | 2020 | MIBS-4 |  |  |  |  |  |  |  |  | Inadequate |
| Hashimoto et al. | 2025 | MIBS-4 |  |  |  |  |  |  | Adequate |  |  |
| Hubig et al. | 2022 | MIBS-4 |  |  |  |  |  |  | Adequate |  |  |
| Igarashi et al. | 2023 | MIBS-4 |  |  |  |  |  |  | Doubtful |  |  |
| Karaci et al. | 2024 | MIBS-4 Turkish | |  | Adequate | Doubtful |  |  |  | Inadequate |  |
| Kaske et al. | 2025 | FAMI |  |  |  |  |  |  | Doubtful |  |  |
| Klan et al. | 2022 | FAMI |  | Very good | Very good |  |  |  | Adequate |  |  |
| Lampl et al. | 2016 | Eurolight |  |  |  |  |  |  | Inadequate |  |  |
| Lampl et al. | 2024 | MIBS-4 |  |  |  |  |  |  | Adequate |  |  |
| Lipton et al. | 2023 | MIBS-4 |  |  |  |  |  |  |  |  | Very good |
| Malheiro et al. | 2025 | MIBS-4 Portuguese | | Adequate | Very good |  |  |  | Adequate | Inadequate |  |
| Matsumori et al. | 2022 | MIBS-4 |  |  |  |  |  |  | Doubtful |  |  |
| Pascual et al. | 2023 | MIBS-4 |  |  |  |  |  |  | Doubtful |  |  |
| Pozo-Rosich et al. | 2025 | MIBS-4 |  |  |  |  |  |  |  |  | Very good |
| Sánchez-Huertas et al. | 2025 | MIBS-4 |  |  |  |  |  |  |  |  | Adequate |
| Sandoe et al. | 2021 | MIBS-4 |  |  |  |  |  |  | Inadequate |  |  |
| Sarkar et al. | 2025 | MIBS-4 |  |  |  |  |  |  | Doubtful |  |  |
| Shapiro et al. | 2024 | MIBS-4 |  |  |  |  |  |  | Doubtful |  |  |
| Sotero et al. | 2025 | MIBS-4 |  |  |  |  |  |  | Doubtful |  |  |
| Takizawa et al. | 2025a | MIBS-4 |  |  |  |  |  |  | Doubtful |  |  |
| Takizawa et al. | 2025b | MIBS-4 |  |  |  |  |  |  | Doubtful |  |  |
| Vernieri et al. | 2025 | MIBS-4 |  |  |  |  |  |  |  |  | Adequate |
